# QSAR, molecular docking, molecular dynamics and DFT-based design of novel Quinoline-2-yl (piperazin-1-yl) inhibitors of hepatitis C virus

**DOI:** 10.1016/j.namjnl.2025.100037

**Published:** 2025-07-11

**Authors:** Abubakar Sadiq Bello, A. Uzairu, G.A Shallangwa, A. ibrahim, Mujeeb Khan, M.T. Ibrahim

**Affiliations:** a Department of Chemistry, Faculty of Physical Science, Ahmadu Bello University, P.M.B 1045, Zaria, Kaduna State, Nigeria; b Department of Chemistry, Faculty of Science, Air force Institute of Technology, P.M.B 2104, Kaduna State, Nigeria; c Department of Pharmaceutical Chemistry, R. C. Patel Institute of Pharmaceutical Education and Research, Shirpur, 425405, Maharashtra, India

**Keywords:** Hepatitis C, NS-5B Polymerase, ADMET properties, Molecular docking

## Abstract

Liver cirrhosis and hepatocellular cancer are brought on by the hepatitis C virus (HCV). Since the virus's discovery, significant therapeutic advancements have been made. Recent studies have demonstrated the growing importance of plant compounds in the creation of novel, efficient, and reasonably priced anti-Hepatitis C virus therapies. The present study involved a thorough analysis of 35 Quinoline-2-yl (piperazin-1-yl) compounds. These compounds were computationally analyzed using in-silico methods like 2D-3D QSAR modeling and molecular docking, and their results were verified through the use of Density Functional Theory (DFT) calculations and ADMET characteristics assessment. The inhibitors were optimized using DFT based on a notion of B3LYP/6–31G* levels. The genetic function algorithm (GFA) was utilized to create the QSAR models. The best model was chosen based on its statistical fitness using the subsequent measurement parameters: R^2^trng = 0.8328, R^2^adj = 0.798036, Q^2^cv = 0.765153, and R^2^test = 0.6510 led to its selection and publication. A reliable 2D QSAR model was constructed and validated using an external validation test set, Y-randomization, variance inflation factor (VIF), mean effect (MF), and William's plot applicability domain (AD). The virtual screening of the compounds under study revealed that compound 29 was the top hit, with the highest mole dock score of -144.45 kcal/mol. A structure-based method was used to create six novel compounds with a higher affinity (SFBD3 -152.299 kcal/mol) for the NS5B polymerase. The food drug administration (FDA) drug Rib/Pig (-111.095 kcal/mol) has lower mole dock scores than the newly created compounds. Molecular dynamics (MD) simulations evaluated the binding stability and dynamic behavior of compounds 29 and SFD B3 with HCV NS5B polymerase (PDB ID: 3QK7) over 100 ns, using Desmond. Analyses of RMSD, RMSF, and hydrogen bonding revealed stable binding for both compounds. SFD B3 demonstrated superior stability (avg RMSD 1.92 Å, 2.91 H-bonds) compared to compound 29 (avg RMSD 2.32 Å, 0.94 H-bonds), indicating more favorable interaction dynamics within the NS5B active site. According to the synthetic accessibility score, the newly discovered compounds may be readily made in a wet lab, have excellent pharmacokinetic profiles, and have a bioavailability score of 0.55 based on the anticipated drug-likeness and ADMET properties. As potential Hepatitis C viral agents, the created molecule SFBD3, which is stable, has a higher affinity, and has the best pharmacokinetic characteristics, ought to be made in a wet lab.

## 1.0 Introduction

Around 170 million people globally are infected with the hepatitis C virus (HCV), that may lead to up to 350,000 deaths annually and has a severe effect on health (Hundt et al., 2015; Manns et al., 2017). HCV infections constitute a preposterous illness that many individuals are uninformed of having as they usually show no symptoms in their initial stages. Chronic liver disease, cirrhosis, and hepatocellular carcinoma are more common in people infected with HCV (Hanafiah et al., 2013). A chronic infection occurs in 50–80 % of newly infected HCV patients; of those, about 30 % develop liver cirrhosis, and up to 4 % develop end-stage liver disease and potentially fatal hepatocellular carcinoma (McCombs et al., 2014). Combining ribavirin and pegylated interferon is the standard treatment for HCV infection. There are many adverse effects, some of which are severe, and this takes 24 to 48 weeks. Additionally, only 50–70 % of patients respond to the regimen, depending on their genotype and clinical stage (McHutchison et al., 2009).

The current standard of treatment for HCV has significantly improved with the approval of several small-molecule direct-acting antiviral (DAA) medications. These medications target viral proteins such NS5B polymerase, NS5A, and NS3/4 A protease that are important in HCV replication (McConachie et al., 2016). To treat individuals with genotype 1 or genotype 4 HCV infection, a newly approved combination of hepatitis C medications (Pak: ombitasvir + paritaprevir + ritonavir + dasabuvir and Zepatier: elbasvir + grazoprevir and Viekira) has also shown increased safety and effectiveness (Keating, 2016). Although there is a new possibility for treating HCV infection thanks to these medications, issues with drug resistance, low genotype specificity, a lack of vaccinations, and high cost still exist (Hill and Cooke, 2014; Lam and Salazar, 2016). It is crucial to discover new anti-HCV drugs, especially ones with unique chemical structures and mechanisms of action (MOAs). N-(4-(dimethyl amino) phenyl)-4‑methoxy-3-propionamidobenzamide molecules have been the subject of ongoing research due to the various roles they play in biological processes and pathologies, including viral infection and (Schroeder et al., 2014) bacterial infection (Brackett et al., 2014).

The 3D-quantitative structure activity relationship (QSAR) method of computer-aided drug synthesis plays a major role in predicting the biological activities of small molecules that have not yet been created (Abdulfatai et al., 2019). Predicting the biological actions of tiny compounds that haven't been produced is made possible in large part by the 3D-QSAR technique of computer-aided drug creation (Abdulfatai et al., 2019). Additionally, by helping to identify the most promising candidates, 3D-QSAR modeling saves a significant amount of time and money by reducing the number of compounds that need to be synthesized throughout the lengthy stage of drug development. Medicinal chemists are now interested in examining the connections between structural characteristics and biological activity as a result of 3D-QSAR's numerous triumphs (Verma et al., 2010).

To identify potential hit candidates, massive datasets of substances are examined using a molecular modeling technique called virtual screening (Yoo et al., 2012). The three-dimensional structures of a ligand and a receptor interact, and the degree of ligand-receptor binding strength is examined, using a different molecular modeling technique known as molecular docking. It will also support virtual compound screening in the pre-clinical stage of medication development (A. Bello et al., 2018) (A. Bello et al., 2019).

Evaluation of absorption, distribution, metabolism, and excretion (ADME) should be carried out earlier in the process of discovery, especially if there are several compounds to consider, because physical samples are not always readily available for drug development (Daina et al., 2017). For drug research and development, it was essential to forecast a small molecule's ADME properties and drug-likeness in lead optimization and hit-to-lead programs (Ferreira and Andricopulo, 2019). This study intends to forecast the drug-likeness and ADME characteristics of those anti-hepatitis C derivatives, in addition to the in silico activity of a few anti-hepatitis C agents and the nature of their interactions with Biaryl amide derivatives via docking, using the QSAR technique (A. S. Bello et al., 2018).

Fighting viral infections requires the creation of novel antiviral medications. A serious worldwide health issue, hepatitis C virus (HCV) underscores the need for improved treatment approaches. Since NS5B polymerase is an essential enzyme for HCV replication, it is a viable target for the creation of antiviral drugs (A. S. Bello et al., 2025). Investigating the structural and functional properties of potential NS5B polymerase inhibitors using computer-aided molecular modeling can speed up the drug discovery process and increase the potency and selectivity of new drugs (A. S. Bello et al., 2024).

The main goal of the study was to use a high-through computational exploration technique to find an interesting, safe antagonist therapy for autoimmune disorders. With that goal in mind, this study made an effort to investigate the issue using computational methods, including the QSAR, design, ADMET analysis, DFT calculations, and research on molecular docking. Furthermore, the results offer empirical data to support further validation, modification, and improvement of the suggested drugs for immune-mediated diseases (Bouayad et al., 2024).

To select new Quinoline-2-yl (piperazin-1-yl) candidates for anti-Hepatitis C drugs, major pharmaceutical companies are increasingly focusing on innovation by adopting new research methodologies and advancing the development of novel compounds (Zerrouk et al., 2025). Molecular modeling studies, such as molecular docking, molecular dynamics, and ADMET analysis, are essential tools for identifying and predicting the efficacy of these new drug candidates (Lenda et al., 2025).

The development of new antiviral medicines is critical in battling viral infections. Hepatitis C virus (HCV) is a major global health concern, highlighting the need for better therapeutic interventions. NS5B polymerase is a critical enzyme in HCV replication, making it a promising target for antiviral medication development. Computer-aided molecular modeling is a valuable tool for investigating the structural and functional characteristics of putative NS5B polymerase inhibitors, expediting the drug discovery process and improving the potency and selectivity of novel compounds. It has been anticipated that the discovery will stimulate further investigation into the immunomodulatory properties of these substances, which go beyond their direct influence on hepatitis illnesses (Er-rajy et al., 2025).

## 2.0 Equipment and techniques

### 2.1 Chemical identification for in-silico investigation

A set of 35 chemical compounds (binding agents) that contained Quinoline and had inhibitory effects on the protein NS5B polymerase were taken out from (Kaur and Kumar 2021). The EC50 values (provided in µm) and the minimal concentration of inhibition were transformed into pEC50 using an inverse logarithmic (pEC50 = -log10 EC50) in order to quickly use the QSAR technique. Later on, the translated variables became variables that relied on the QSAR analysis. Details about each ligand, such as its molecular formula, pEC50 values, and residual values, are listed in Table 1.

**Table 1:** The Molecular structure, experimental EC50, predicted pEC50, of some Quinoline-2-yl (piperazin-1-yl) derivatives against NS 5B polymerase.

S/N	Structure	Activity EC_50_ (µm)	PEC_50_(µm)
1		0.57	6.244125
2		0.07	7.154902
3		0.46	6.337242
4		2.27	5.643974
5		1.16	5.935542
6		0.07	7.154902
7		0.06	7.221849
8		0.05	7.30103
9		0.09	7.045757
10		0.80	6.09691
11		0.10	7.000
12		0.02	7.69897
13		0.05	7.30103
14		0.14	6.853872
15		0.05	7.30103
16		0.98	6.008774
17		0.67	6.173925
18		0.22	6.657577
19		0.56	6.251812
20		0.25	6.60206
21		0.22	6.657577
22		0.26	6.585027
23		0.75	6.124939
24		0.86	6.065502
25		2.01	5.696804
26		0.97	6.013228
27		0.31	6.508638
28		3.09	5.510042
29		0.84	6.075721
30		1.88	5.725842
31		9.20	5.036212
32		0.57	6.244125
33		0.07	7.154902
34		0.46	6.337242
35		2.27	5.643974

### 2.2 Calculating stable geometry and generating structures

Following data collection, the next stage of this study is to draw the dataset's 2D structures. The 2D structures of the dataset were produced using Chemdraw 12.0 (Ibrahim et al., 2018). The Spartan 14 program automatically converted the two-dimensional configurations of the molecules under study from 2D to 3D before energy minimization. Energy minimization resulted in less constraint in the structures before using the same software to determine the most stable geometry of the molecules under study on the potential energy surface. Using Density Functional Theory at the B3LYP/6–311G* level and the Theory of Global Minima on the Potential Energy Surface (PES), the strongest configurations of every molecule under investigation were found (Kohn et al., 1996).

### 2.3 Data pre-treatment, dataset partitioning, and the creation of descriptors

The descriptors, or independent variables, were computed using the PaDEL descriptor tool set. It performs 1D, 2D, and 3D descriptor computations (Yap, 2011). To eliminate redundant and consistent descriptions, the dataset was thoroughly pre-treated. Using data division software, the pre-treated data were subsequently separated into a training set and a test set using the Kennard-Stone technique (Kennard and Stone, 1969).

The training set and the test set were separated in an 80:20 ratio from the data set, which included the chemical component, in order to generate the QSAR models. The training set, which comprises 80 % of the chemical compounds under consideration, is used to create the QSAR model. Although the test set—which comprises the final 20 % of the entire chemical compound data set—was used to assess the analytical quality of the established model rather than to develop the QSAR model, (Hill and Cooke, 2014).

### 2.4 Model development

Models were created using the real pEC50µM as the response variable and the Genetic Function Approximation (GFA) approach. The independent variables used were descriptors. The capacity of GFA to choose the most closely related descriptors from which to generate a large number of models is one of its unique characteristics. The Genetic Function Algorithm (GFA) in Material Studio software version 8 was used to establish the internal validation criteria. There were five descriptors in the regression equation, and the values for Population and Generation were 1000 and 1500, respectively. Friedman's Lack of Fit (LOF), which gauges a model's fitness score, was used to grade the models. The following is the updated LOF formula:

(1) $$\text{LOF}=\frac{\text{SSE}}{\frac{1-\;C+\text{dp}}{M}}$$

where M is the number of training set samples, d is a user-defined smoothing parameter, p is the total number of descriptors present in all model terms (excluding the constant term), c is the number of terms in the model other than the constant term, and SSE is the sum of squared errors (A. Bello et al., 2018)..

### 2.5 Selecting and validating the model

Given the square correlation coefficients (R^2^ test and R^2^ training) of the training and test sets, which are all stated as equations, the most commonly used statistic for evaluating QSAR models is the cross-validation coefficient (Qcv^2^), also known as adjusted R^2^ (R^2^ adj). (3–6). Despite appearing to be necessary, these elements' high values are insufficient (Bajorath, 2015). Eq. (7) is used to compute the coefficient of determination, or cR^2^p, for Y-randomization. R^2^ is the average "R" of random models, and cR^2^p is the coefficient of determination for Y-randomization. This metric must be >0.5 in order to pass this test. To be deemed reliable and resilient, a built QSAR model needs to have low R^2^ and Q^2^ values across several trials.

(2) $$R^{2}=1-\frac{\sum {\left(Y_{obs}-Y_{pred}\right)}^{2}}{\sum {\left(Y_{obs}-{\bar{Y}}_{training}\right)}^{2}}$$

(3) $${R^{2}}_{adj}=1-\left(1-R^{2}\right)\frac{N-1}{N-P-1}=\frac{\left(N-1\right)\;R2-P}{N-P+1}$$

(4) $${Q^{2}}_{cv}=1-\frac{\sum {\left(Ypred-Y\exp\right)}^{2}}{\sum {\left(Y\exp-Ymtraining\right)}^{2}}$$

(5) R^2^_pred_=1−Σ(Y_pred_–Y_exp_)/Σ(Y_exp_ – Y_mntrng_)

(6) cR^2^p = *R* × (R^2^ − R^2^r) ^2^

### 2.6 The mean effects (MF) and variation in inflation factors VIF

Each descriptor's positional impact on the model was evaluated using its Mean effect value. The Mean impact is explained by equation (I).

(7) $$MF=\frac{\beta_{j}\sum_{i=1}^{i=n}d_{ij}}{\sum_{j}^{m}\beta_{j}\sum_{i}^{n}d_{ij}}$$

For each molecule in the evolving set of the framework, the characteristic's accumulation in the information point, the descriptive variable j in the model's structure, and the overall number of descriptors in the technique are indicated by the symbols Bj, dij. m., and n (Ejeh et al., 2023).

By calculating the variance of inflation factors (VIF), which will be evaluated in the manner described below, the presence of multi-collinearity among the descriptors was discovered:

(8) $$\text{VIF}=\frac{1}{1-R^{2}}$$

R^2^ is a measure of similarity during multiple regression analysis, specifically between model variables.

### 2.7 Domain of applicability

After passing the application domain (AD) test, a QSAR model is considered valid and unreliable if it is able to predict novel behaviors of the training and testing molecules with accuracy. The reported model was run through AD to see if the molecules under examination contained any noteworthy or unusual compounds (Eriksson et al., 2003). One method for assessing the AD is the leverage approach, which has the following advantages:

(9) h_i_ = x_i_ (X^T^ X)^_K^ x_i_^T^ (i = A,…, Z)

Here, X stands for the training set's *n* × *k* descriptor matrix, XT for the transpose matrix X that was used to create the model, and xi for the training set matrix I. The following equation provides the threshold-hold for the value of X, which is the warning threshold (h*).

(10) h* = 3(*x* + 1)/q ………………….

Here, the number of compounds in the model building set is denoted by q, and the number of descriptors in the model under evaluation is represented by x.

### 2.8 Completion and evaluation of the molecular docking simulation

Using the Molegro Virtual Docker (MVD), thirty-five (35) sets of geometry-optimized Quinoline-2-yl (piperazin-1-yl) compounds were created by selecting "if missing" from the list of choices the software uses to construct compounds and ligands (Ibrahim et al., 2020). The NS5B polymerase protein database (https://www.rcsb.org/) was used to get the protein of interest (NS5B polymerase enzyme with entry symbol 3FQK) as a complex. Additionally, the NS5B polymerase was created and its active/binding sites/poses were discovered before ligand-receptor docking by evaluating the recovered crystalline structure using Discovery studio visualizer version 16.1.0.15350.

Plant values as a docking strategy and mole dock values as an assessment tool were used in the molecular docking procedure. The grid box size was changed to 24 Armstrong units in order to identify the binding spaces in the protein's binding postures. Consequently, the default parameters for all other computations within the processes were maintained (Abdullahi et al., 2020).

### 2.9 Structure-based approach

A structure-based modeling approach was used in this investigation. Structure-based synthesis has been acknowledged as a quick way to create medications that are used in situations when there is little information available regarding the receptors and targets that are needed. Finding the best hit within the compound collection under study can be achieved by combining fragment-based virtual evaluation with molecular docking simulation. The molecule found to be a hit after a thorough molecular docking virtual evaluation will be retained as a starting molecule and changed or modified to produce new compounds with a greater affinity for the NS5B polymerase than the ones that were previously found.

### 2.10 MD simulation

To evaluate the binding stability of compounds 29 and SFD B3 within the active site of NS5B polymerase (PDB ID: 3QK7), molecular dynamics (MD) simulations were performed using the Desmond Molecular Dynamics System (version DESMOND/G 6.9.137). Simulations were carried out on a high-performance Linux-based workstation equipped with an Intel® Xeon® W-2245 CPU @ 3.90 GHz × 16, NVIDIA RTX A4000 GPU (GA104GL architecture) with CUDA 12 support, running Ubuntu 22.04.2 LTS. The systems were prepared using the System Builder tool in Desmond, solvated in an orthorhombic box using the TIP3P water model with a buffer distance of 10 Å, and neutralized with appropriate counter-ions. Prior to initiating the simulation, the protein-ligand complexes were subjected to energy minimization to eliminate steric clashes and relax any unfavorable electronic interactions, thereby ensuring a stable starting conformation(Girase et al., 2024). The systems were equilibrated using the default relaxation protocol provided in Desmond, which includes a series of restrained and unrestrained minimization and short MD steps to allow temperature and pressure adaptation. Production MD simulations were run for 100 ns in the NPT ensemble, maintaining a constant temperature of 300 K using the Nose-Hoover thermostat and a pressure of 1.0 bar using the Martyna-Tobias-Klein barostat (Singh et al., 2025).

### 2.11 Density functional theory (DFT) computations

We performed DFT/B3LYP level calculations on the examined compounds in order to investigate the structural geometries. A key method for measuring molecule reactivity and studying the stimulation of electrons spanning the most highly occupied molecular orbital (HOMO) to the least unoccupied molecular orbital (LUMO) is to look at the frontier orbitals of molecules in addition to geometric optimization. While the HOMO shows the molecule's ability to give away electrons, the LUMO establishes the molecule's ability to collect electrons. The difference in energy content between the HOMO (EH) and LUMO (EL) orbitals is known as the HOMO-LUMO gap in energy (DE), and it characterizes the charge transfer interaction process that occurs inside the molecule's structure. We might be able to comprehend our compounds' kinetic and chemical reactions better if we had a link to the energy gap (DE). In actuality, molecules with a narrow energy gap are typically categorized as soft because they have limited kinetic strength and high chemical reactivity.

Conversely, hard compounds are more stable because they have a larger energy gap. This results from variations in electron density and dispersion, as well as their resistance to charge transfer (Fukui, 1982). Additionally, several other characteristics, such the ionization potential IP (IP = -EH) and the electron affinity EA (EA = -EL), which are closely linked to the HOMO and LUMO energies, could be used to describe a larger number of our particles (Wolinski et al., 1990). Using these properties, we can compute the following (Gázquez, 1993). The formula for global hardness (ƞ) (and its inverse, σ global softness) indicates a molecule's polarizability.

(3a) $$Ƞ=\frac{IP-EA}{2}$$

Electronegativity (ꭓ) refers to a molecule's ability to gather electrons and is represented by the following formula:

(4a) $$\chi =\frac{IP+\;EA}{2}$$

The chemical potential (μ) is the initial component of the whole energy having regard to the number of electrons in a molecule, showing an electron's willingness to escape (El Kalai et al., 2019). The chemical potential is just the opposite of the electron attraction worth:

(5a) μ=-ꭓ ………

The electrophilicity index (ω) is a measure of a substance's ability to take electrons and was proposed to assess its electrophilic power. The formula states:

(6a) $$\omega =\frac{\mu^{2}}{2ƞ}$$

Table 6 lists the computed proprieties. These findings indicate a significant difference in EH, EL, and DE.

### 2.12 Pharmacokinetics feature evaluation

Early assessment of pharmaceutical molecules' effects on biological procedures is essential in today's drug development and discovery process in order to weed out non-drug-like compounds from the group of physiologically active molecules. The biological method's processes of absorption (A), distribution (D), metabolism (M), excretion (E), and toxicity (T) (ADMET) dictate the future of potential drug candidates. The pKCSM (http://biosig.unimelb.edu.au/pkcsm/) web platforms were used to assist in the ADMET assessment of the discovered bioactive compounds. SwissADME (www.swissadme.ch/), a web-based server, was used to investigate the drug-like responses of the chosen chemicals.

## 3.0 Findings and discussions

To determine the activities and characteristics of quinoline-2-yl (piperazin-1-yl) compounds as efficient hepatitis-C virus NS5B protease inhibitor agents, a QSAR technique utilizing multiple algorithms was developed; Table 2 displays the QSAR model. Table 2 shows the results of this model's development and evaluation using QSAR software. Many suitable model criteria are used to evaluate the model. 84 % and 90 % of the changes in quinoline-2-yl (piperazin-1-yl) compounds with anti-hepatitis-C virus bioactivity towards HCV NS5B polymerase are described by the suggested model. Table 2's simulated statistics results satisfy the requirements for assessing a QSAR model.

**Table 2:** shows the verification parameters used in the models developed using the suggested values for evaluating an approved QSAR model.

Parameters	Model 1	Model 2	Model 3	Model 4	**Model 5**
Friedman LOF	0.307879	0.309483	0.309801	0.312408	0.312540
R-squared	0.835350	0.834493	0.834322	0.832929	0.832858
Adjusted R-squared	0.801048	0.800012	0.799806	0.798122	0.798036
Cross validated R-squared	0.765180	0.759019	0.764059	0.757234	0.765153
Significant Regression	Yes	Yes	Yes	Yes	Yes
Significance-of-regression F-value	24.35281	24.201718	24.171923	23.930225	23.91804
Critical SOR F-value (95 %)	2.64405	2.64405	2.64405	2.64405	2.64405
Lack-of-fit points	23	23	23	23	23
External Validation	0.585109	0.602201	0.580764	0.597856	**0.651095**

Model 5 PEC_50_μm = 0.000202273 * ATS5i - 3.036142922 * ATSC7c + 0.001029243 * ATSC5v + 52.496826932 * AATSC - 14.248882100 * SpMin6_Bhm - 23.52388052

Thus, Q^2^ is the squared cross-validation coefficients after excluding one, and K is the model's predictor variables (descriptors), whereas R^2^train and R^2^test were the correlation coefficients for internal and external validation, respectively. Considering the aforementioned findings, the model's R^2^ External Validation of 0.6510 indicates that it provides a good fit for emulating anti-HCV inhibitory functions. The model's resilience and efficiency satisfy the conventional evaluation standard. According to the y-randomization evaluation results, the model is rarely the product of pure coincidence, as evidenced by the permutation R^2^ (cR^2^r = 0.7283), which is much higher than the minimal value of 0.50 (Table 2).

According to Eq. (8), there is no inter-correlation between the variables whenever the VIF is equal to 1. When VIF falls between 1 and 5, the accompanying model is deemed appropriate. However, the model becomes unstable and needs to be reevaluated when VIF rises over 10. Table 3 displays the VIF values for each of the five (5) descriptors. The created model is statistically significant, and the descriptors are compatible, as the table demonstrates, with each descriptor having a value of VIF 1–3. The mean effect (MF), which illustrates a descriptor's relative significance to the other descriptors in the model, is also displayed in Table 3. Its polarity indicates the direction that the variables in the computations take as a result of an increase or decrease in descriptor values.

**Table 3:** Pearson’s correlation matrix, VIF and Mean effect of the descriptors in model 5.

	ATS5i	ATSC7c	ATSC5v	AATSC	SpMin6_Bhm	*VIF*	ME
ATS5i	1					2.46933	3.50073
ATSC7c	−0.2345	1				1.91880	1.00685
ATSC5v	−0.6549	0.456281	1			3.38420	2.51482
AATSC	0.06805	−0.73802	−0.13405	1		2.04016	−1.00804
SpMin6_Bhm	0.49389	−0.50071	−0.42009	0.282022	1	2.43328	−2.01437

Table 4 shows the results of the Y-scrambling Test for ten randomly built models [10]. It was revealed that the newly generated random models had poor R^2^ and Q^2^ values. This proves that the claimed model's availability was not a coincidence and adds to the model's legitimacy.

**Table 4:** Y-randomization.

Model	R	R^2	Q^2
Original	0.868219	0.753804	0.657365
Random 1	0.200392	0.040157	−0.42723
Random 2	0.213521	0.045591	−0.42001
Random 3	0.342764	0.117487	−0.22266
Random 4	0.401167	0.160935	−0.39556
Random 5	0.41361	0.171073	−0.17587
Random 6	0.423323	0.179202	−0.15987
Random 7	0.30317	0.091912	−0.40702
Random 8	0.280194	0.078509	−0.36532
Random 9	0.342794	0.117508	−0.41624
Random 10	0.346239	0.119882	−0.2475
Random Models Parameters			
Average r:	0.326717		
Average r^2:	0.112226		
Average Q^2:	−0.52373		
cRp^2:	0.728396		

**Fig. 1:**
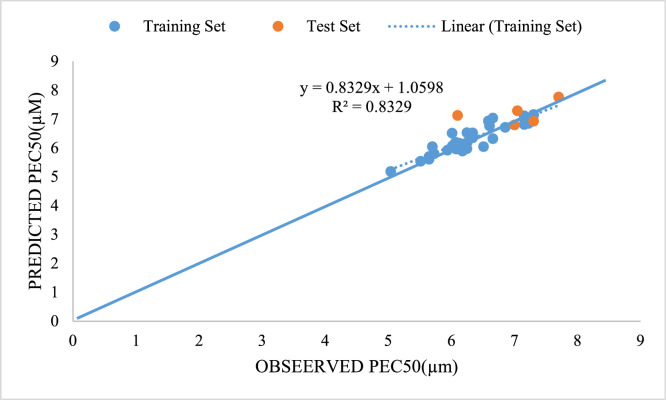
XY (Scatter) Plot of the actual pEC_50_ against predicted pEC_50_ of training set and test set of the selected model.

**Fig. 2:**
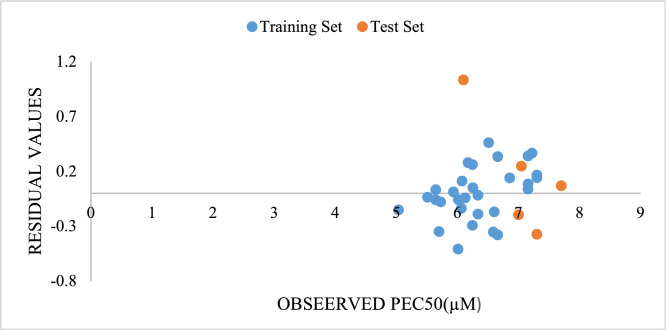
XY (Scatter) Plot of actual pEC_50_ against the residuals of both the test and training sets of the selected model.

Fig. 1 depicts the actual plot of experimental vs. anticipated outcomes for the confirmed GA-MLR method. This graph shows that the majority of the datasets were somewhat distributed along the line of best fit, implying that the correlation indicates a strong association between the investigated bioactivity and the two descriptors. Fig. 2 also showed a scatter plot of residual and projected values, as well as the model residual's dispersion over the standardized residual, which was zero. As practical means, the models were discovered to have a high predictive potential and be free of systemic bias. As a result, as long as a molecule exists inside the models' applicability domain (AD), it is frequently utilized to assess the activity of recognized compounds.

**Fig. 3:**
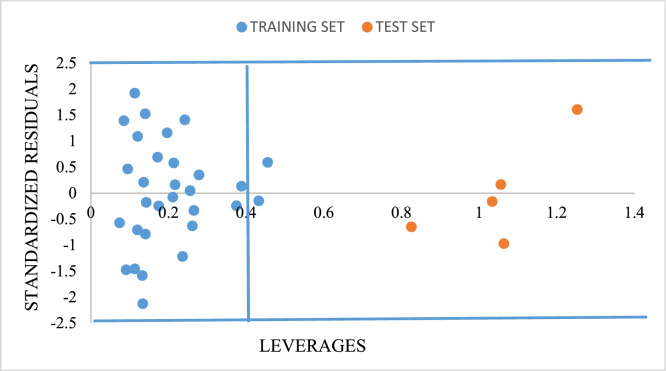
Williams Plot of the selected model.

The assigned value for h* in this model is 0.404. Should the leverage exceed the warning/cautionary leverage h*, the anticipated outcome might not be accurate. All of the compounds from the test sets and one of the two compounds in the training set have amplitudes of hi scores that are greater than the predefined warning threshold value, as can be observed in the Williams plot that depicts this QSAR model (Fig. 3). Numerous compounds have notable impacts on the model area, making the training set more than adequate. The fact that compounds with leverage values greater than the h* threshold are not taken into account while developing new hepatitis C drugs, however, must be understood. The actuation mechanisms used to construct the described model may differ structurally from those of these molecules.

### 3.1 DFT computations

We used DFT/B3LYP level calculations to undertake full geometry optimization for the compounds under study in order to examine the structure geometries (Table 5). A compound's long-term viability can be understood by using its frontier molecular orbital energies (HOMO and LUMO energies), which indicate how important its properties are in relation to connections connected to the transfer of charge alongside other molecules (such as at the active site of proteins or enzymes). A molecule's electrical and optical characteristics, which indicate its reactivity, can be influenced by its energy band gap (ΔE) (Ibrahim et al., 2024). Furthermore, the energy gap makes it possible to identify really powerful compounds among the top hits. Additionally, the compounds' bioactivities and intermolecular charge transmission from HOMO to LUMO were significantly impacted by the smaller energy difference between their HOMO and LUMO energies, which led to a larger affinity of the compounds for the target enzyme (NS5B polymerase). The compounds' bioactivities and intermolecular transfer of charge from HOMO to LUMO are negatively impacted by a greater energy differential between the HOMO and LUMO energies of the most effective hit, which results in a slight attraction of the compounds toward the targeted protein (Ibrahim et al., 2024). The lowest potential and lowest band/energy gap (ΔE) of 4.13 eV are found in compound number five, the greatest hit (see Table 5). Because of its smaller band difference, it may have the strongest attraction for the NS5B polymerase protein, suggesting that it is the most sensitive of the chemicals reported. Complex development is more stable when band gaps are smaller.

**Table 5:** Frontier molecular-orbital energies and global-reactivity descriptors of the studied ligands.

S/N	EH	EL	DE	IP	EA	ƞ	ꭓ	μ	ω
5	−5.13	−1	−4.13	5.13	1	4.63	5.63	−4.63	49.62642
19	−5.42	−1.11	−4.31	5.42	1.11	4.865	5.975	−4.865	57.57296
35	−5.36	−1.05	−4.31	5.36	1.05	4.835	5.885	−4.835	56.51444
8	−5.4	−1.02	−4.38	5.4	1.02	4.89	5.91	−4.89	58.46508
34	−5.43	−1.05	−4.38	5.43	1.05	4.905	5.955	−4.905	59.00476

**Fig. 4:**
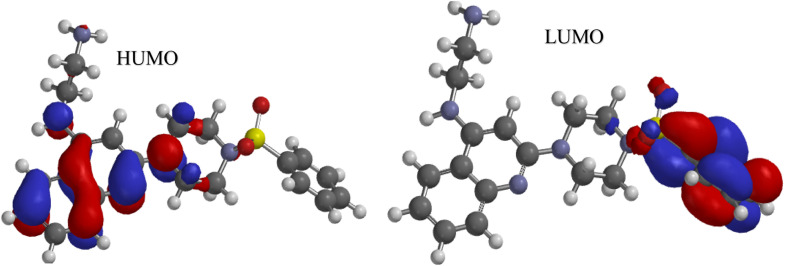
Shapes of MOs frontiers of compound 5 obtained by DFT (B3LYP/6–31G*) method.

In addition, a compound's reactivity changes in tandem with the band gap between HOMO and LUMO energies. The highest hit's responsiveness rises in the order listed below: Compounds 5 (4.13 eV) > 19 (4.31 eV) > 35 (4.31 eV) > 8 (4.38 eV). Quantum chemical properties (η, δ, χ, μ, and ω) have been calculated using the compound's HOMO and LUMO energies, and compound 1′s stability, reactivity, and visual properties have been confirmed (refer to Table 6). The stability and reactivity of molecules are related to chemical hardness (η). A harder atom will have a larger band gap, which means the molecule will be less reactive. A softer protein, on the other hand, has a smaller band gap. The frontier molecular orbital (FMO) energy maps of the most promising molecule, commonly referred to as HOMO and LUMO, are displayed in Fig. 4.

**Table 6:** MolDockScore, Plants Score, RerankScore of the Quinoline-2-yl (piperazin-1-yl) Compounds.

Name	MolDock Score kcal/mol.	Heavy Atoms	Plants Score	Rerank Score
Compound 1	−81.573	24	−65.257	−53.710
Compound 2	−99.763	29	−75.730	−89.050
Compound 3	−93.927	29	−76.072	−81.078
Compound 4	**−120.96**	29	−83.706	−100.19
Compound 5	−106.77	29	−79.370	−88.198
Compound 6	−86.540	29	−84.127	−75.961
Compound 7	−117.69	29	−85.794	−100.86
Compound 8	−94.359	29	−76.560	−82.912
Compound 9	−112.76	29	−85.064	−96.886
Compound 10	−112.25	29	−78.744	−64.486
Compound 11	−93.018	29	−80.092	−81.098
Compound 12	−112.79	30	−82.230	−97.578
Compound 13	−119.54	30	−80.535	−101.32
Compound 14	−121.50	30	−80.905	−97.928
Compound 15	−102.21	30	−83.614	−90.830
Compound 16	−100.12	32	−90.215	−89.803
Compound 17	**−142.07**	30	−93.455	−119.71
Compound 18	−99.878	30	−81.525	−86.771
Compound 19	−94.450	30	−82.351	−81.737
Compound 20	−96.127	30	−76.457	−77.943
Compound 21	−94.248	31	−86.809	−75.790
Compound 22	**−128.14**	31	−81.950	−114.02
Compound 23	−91.098	33	−75.044	−63.769
Compound 24	−106.99	35	−79.068	−91.024
Compound 25	−82.981	32	−80.489	−69.684
Compound 26	−104.38	33	−80.181	−88.307
Compound 27	−102.94	34	−89.224	−91.284
Compound 28	−119.22	31	−80.522	−95.607
Compound 29	**−144.45**	32	−78.445	−122.12
Compound 30	−101.14	32	−77.797	−74.410
Compound 31	−114.33	32	−77.520	−91.733
Compound 32	−103.19	25	−72.981	−84.973
Compound 33	−116.92	21	−76.098	−98.214
Compound 34	−113.76	28	−78.382	−93.842
Compound 35	−94.260	31	−80.160	−84.684
**FDA Rib/Pig**	**−111.095**	**37**	**−97.670**	**−46.992**

**Fig. 5:**
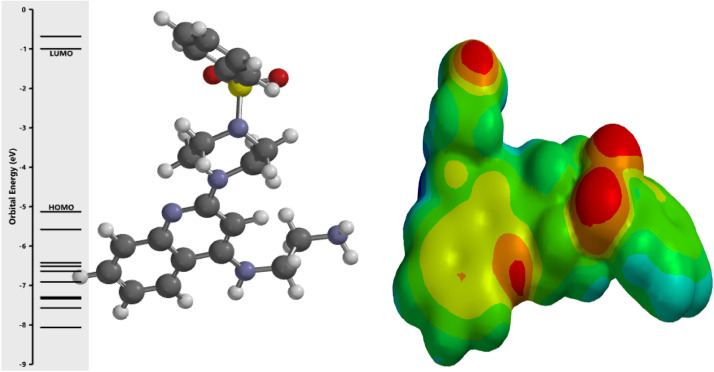
Orbital energy eV and surface map of compound 5 the best hit compound.

A detailed understanding of how the molecule will interact with other molecules, especially the enzyme (NS5B polymerase) in ligand-protein interactions, is provided by the MEP surface allocation map of compound 5, which is based on the polar areas of the surface determined according to quantum chemical characteristics. The MEPS maps for the most effective drug, compound 5, are shown in Fig. 4. The MEP surface for compound 5′s visual structure and magnitude are depicted by the color shades. On the MEPS map, a positive potential zone or region is represented by the color blue, whereas a negative potential zone or region is represented by the color red. Compound 5′s amino groups were dominated by the blue area, which is known as the positive potential zone. On the other hand, the majority of the photos were red, which stands for compound 5′s nitrogen and oxygen atoms' negative potential zone. Furthermore, the greenish color, which stood for the area or zone of zero potential, was mostly scattered close to the carbon atom as shown in Fig. 5.

### 3.2 Implementing virtual screening utilizing docking molecules

Accordingly, a molecular docking-based approach was used to examine the ligand-protein combinations of the thirty-five (35) Quinoline-2-yl (piperazin-1-yl) compounds and NS5B polymerase utilizing the 3QK7 pdb entry codes. Based on mole dock results, the NS5B polymerase inhibitors under investigation were given priority. According to Table 6, the mole dock indicates that the molecules under investigation have a range of −81 to −145 kcal/mol. The top hit among the compounds analyzed by the docking-based virtual screening was compound 29, which had a mole dock score of −144.45 kcal/mol. Compound 17 came in second with a mole dock score of −142.07 kcal/mol. Compound 4 was ranked fourth with a mole dock score of −120.96 kcal/mol, and Compound 22 was ranked third with a mole dock score of −128.14 kcal/mol.

**Fig. 6:**
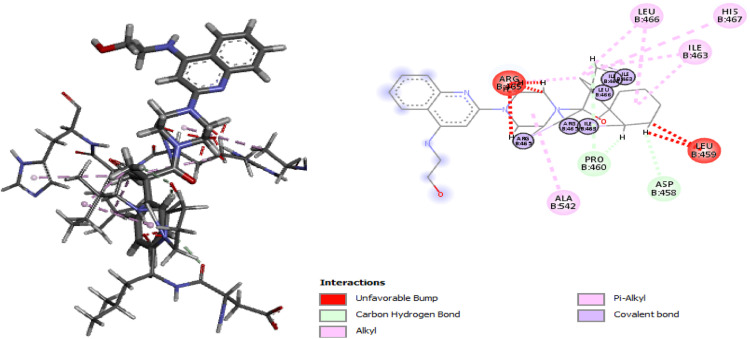
2d, 3d view and hydrogen bond interaction of the componud 29 using dicovery studio virtualizer.

It was discovered that compound 29, the most well-known of the NS5B polymerase inhibitors under investigation, had various unique forms of interactions with the active site of the NS5B polymerase. Fig. 6 shows the Conventional Hydrogen Bond and bond distance between the depend on residues PRO460 (2.70956 Å) and ASP458 (2.36744 Å) amino acids, the Quinoline moiety of the hydrophobic head of compound 29, and the Hydrophobic Alkyl and Hydrophobic Pi-Alkyl interactions between ILE463 (4.35739 Å), LEU466 (5.12798 Å), ALA542 (5.00489 Å), ARG465 (4.34391 Å), LEU466 (3.56984 Å), ILE463 (3.79979 Å) and HIS467 (5.11928 Å) amino acids.

**Fig. 7:**
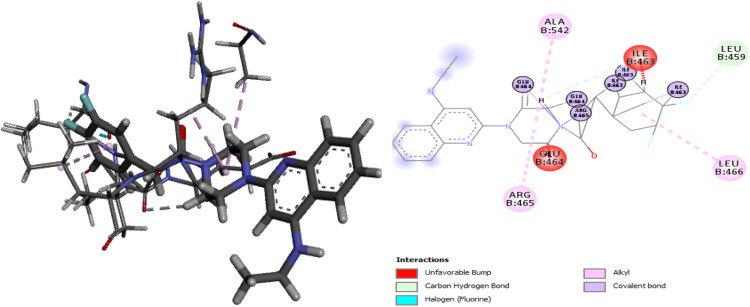
2d, 3d view and hydrogen bond interaction of the componud 17 using dicovery studio virtualizer.

The second-greatest molecule among the Quinoline-2-yl (piperazin-1-yl) agents according to investigation, Molecule 17 within the enzyme site of the NS5B polymerase, has interactions and bond angles through two conventional carbon-hydrogen bonds, LEU459 (2.63347 Å) and ILE463 (2.61189 Å), as well as one hydrogen atom attached to the Quinoline-2-yl (piperazin-1-yl) (at the hydrophobic tail) LEU459 (2.92045 Å), and three hydrophobic Alkyl among ARG465 (3.23716 Å), LEU466 (4.84214 Å), and ALA542 (5.31544 Å) amino acid residues as shown in Fig. 7.

**Fig. 8:**
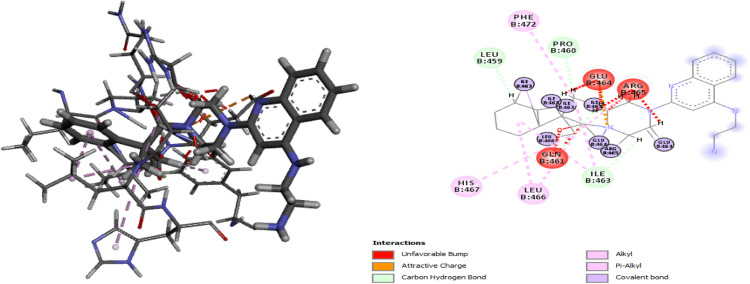
2d, 3d view and hydrogen bond interaction of the componud 22 using dicovery studio virtualizer.

Compound 22, which ranks third in the MolDock Score, PlantsScore, RerankScore, and Heavy Atom rankings, established the Electrostatic Attractive Charge of GLU464 (4.90041 Å), three conventional hydrogen bonds, and one carbon hydrogen bond with their bond angles between ILE463 (2.36891 Å), PRO460 (2.57444 Å), LEU459 (2.17920 Å), and GLN461 (2.72828 Å) residues (at the hydrophobic head) of the molecule, as well as hydrophobic Alkyl and hydrophobic Pi-Alkyl interactions between ILE463 (4.65754 Å), LEU466 (4.63615 Å), ARG465 (4.37455 Å), LEU466 (3.81445 Å), ILE463 (3.65509 Å), HIS467 (5.09051 Å), and PHE472 (5.31323 Å) residues and Quinoline-2-yl (piperazin-1-yl) in the central hetero aromatic system, respectively (Fig. 8).

### 3.3 MD simulation

MD simulation studies were performed to evaluate the binding affinity and dynamic stability of compounds SFD B3 and 29 within the active site of the NS5B polymerase (PDB ID: 3QK7). Docking results revealed favorable binding poses and key interactions, which were further validated through 100 ns MD simulations using the Desmond simulation package. These simulations offered detailed insights into the conformational behavior, protein-ligand stability, and interaction dynamics of both complexes under near-physiological conditions. Comprehensive analyses, including root mean square deviation (RMSD), root mean square fluctuation (RMSF), and hydrogen bonding, were used to assess the stability and binding performance of each compound throughout the simulation trajectory (Boulaamane et al., 2024).

**Fig. 9:**
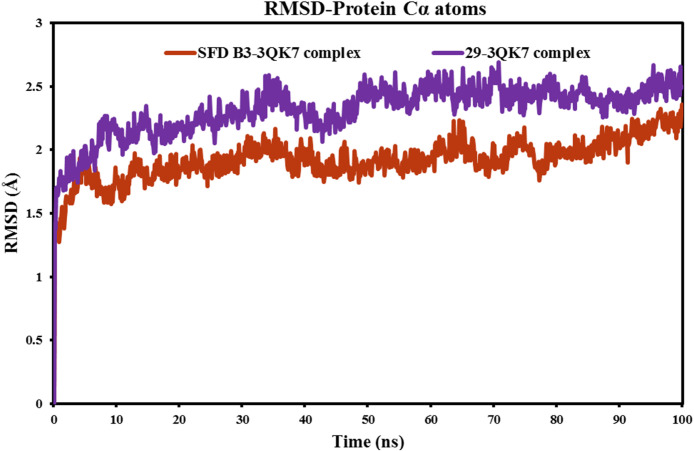
Root Mean Square Deviation (RMSD) trajectories of the NS5B polymerase in complex with compound SFD B3 and compound 29 over a 100 ns simulation period.

RMSD is a widely used metric to assess the structural stability of protein-ligand complexes during MD simulations. It calculates the average positional deviation of atomic coordinates over time, relative to the initial structure. A stable RMSD trajectory generally indicates that the complex maintains its structural integrity, while large fluctuations may suggest conformational instability or weak ligand binding (I. Ahmad and H. M. Patel, 2025). In this study, the RMSD profiles for both SFD B3–3QK7 and compound 29–3QK7 complexes showed consistent and stable behavior throughout the 100 ns simulation. A slight rise in RMSD was observed during the initial 5 ns, attributed to the equilibration phase (Fig. 9). Afterward, both complexes maintained steady trajectories without major deviations. The average RMSD values for the SFD B3 and compound 29 complexes were 1.92 Å and 2.32 Å, respectively, with a maximum deviation of approximately 2.6 Å in both cases. These results indicate that both compounds remained stably bound within the binding cavity, without inducing significant conformational changes in the protein (Saiyad et al., 2025).

**Fig. 10:**
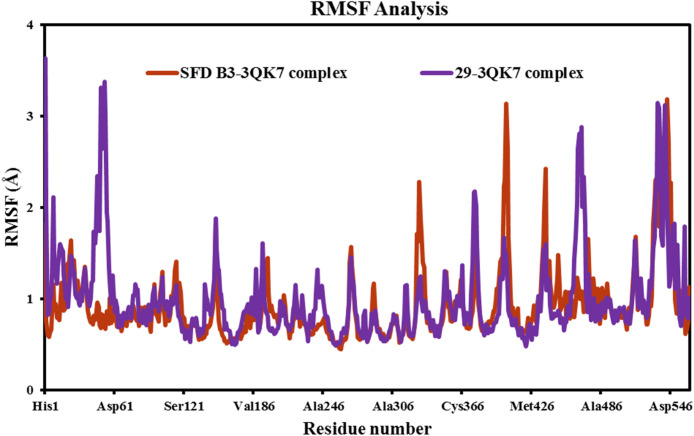
Root Mean Square Fluctuation (RMSF) profiles of the NS5B polymerase residues in complex with SFD B3 and compound 29.

RMSF analysis was conducted to investigate the flexibility of individual amino acid residues upon ligand binding. This parameter is particularly useful for identifying dynamic regions within the protein, such as loops and termini, as well as for evaluating the stability of interacting residues (Chabhadiya et al., 2024). In the current study, both SFD B3–3QK7 and compound 29–3QK7 complexes exhibited low RMSF values across most residues, with average values of 0.93 Å and 1.0 Å, respectively (Fig. 10). Higher fluctuations were observed at Gly46–Lys50, Pro404, and Thr329, regions typically associated with flexible loops and not directly involved in ligand binding. Crucially, all key interacting residues—including Asn35, Gln148, Ser196, Glu202, Lys379, Gln461, Ile462, Ile463, Glu464, Arg465, Leu466, His467, Gly468, Leu469, Ala471, Phe472, Lys535, Leu536, Ala542, Ser543, Leu547, Ser548, Trp550, and Phe551—showed RMSF values below 2 Å, suggesting stable interactions and minimal structural disturbance at the binding site.

**Fig. 11:**
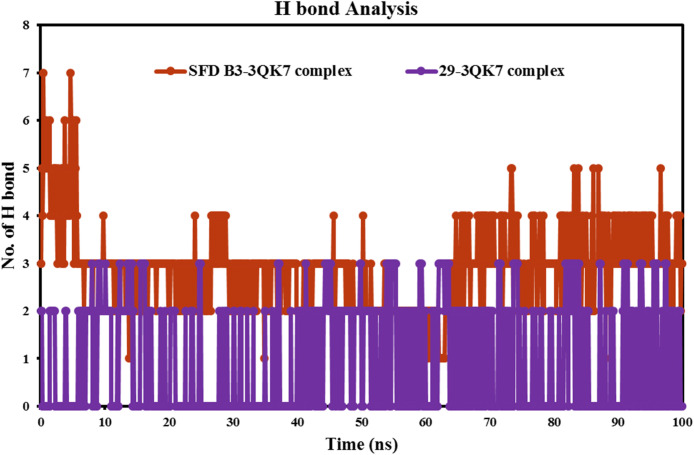
Number of hydrogen bonds formed between NS5B polymerase and compounds SFD B3 and 29 during the 100 ns MD simulation.

Hydrogen bond analysis further confirmed the differential stability of the two complexes. Hydrogen bonds play a critical role in maintaining ligand binding and orientation within the active site (I. Ahmad and H. Patel, 2025). In the SFD B3–3QK7 complex, a maximum of 7 hydrogen bonds was observed, with an average of 2.91 hydrogen bonds consistently maintained throughout the simulation. In contrast, the compound 29–3QK7 complex exhibited a maximum of 3 hydrogen bonds, with an average of 0.94 (Fig. 11). The higher frequency and stability of hydrogen bonds in the SFD B3 complex indicate stronger and more persistent interactions with NS5B polymerase, supporting its superior conformational stability and binding performance. Overall, these results suggest that both compounds exhibit stable binding within the NS5B active site; however, SFD B3 demonstrates more favorable interaction dynamics and structural stability compared to compound 29, making it a more promising candidate for further development.

### 3.4 Modeling and evaluation of pharmacokinetics, including ADME-toxicity and drug-likeness

The drug-likeness of top-ranked hit compounds identified using notable RO5 (MW ≤ 500, Number of HBD ≤ 5, Number of NBA ≤ 10, Calculated MLog *p* < 5 TPSA and ≤140 Å) screening characteristics was examined using SwissADME's web server (Table 6, Table 7). Initially, the most successful, top-ranked hit compounds were screened for drug-likeness using Lipinski's rule of five filtering. Following their filtering using Veber's criterion, it was discovered that each of them had two infractions, indicating that they may all have the capacity to be pharmacologically active. All of the Quinoline-2-yl (piperazin-1-yl) derivatives meet Lipinski's criterion, as shown in Table 7, suggesting that these substances have the highest oral bioavailability.

**Table 7:** Drug-like features of the analogues.

Molecules	MW	H-bond acceptors	H-bond donors	TPSA	Lipinski violations	Synthetic Accessibility
Molecule 1	375.47	3	2	74.49	0	2.82
Molecule 2	389.49	3	2	74.49	0	3.00
Molecule 3	403.52	3	2	74.49	0	3.03
Molecule 4	349.45	5	2	99.94	0	3.07
Molecule 5	411.52	5	2	99.94	0	3.40
Molecule 6	408.92	2	1	48.47	0	3.04
Molecule 7	408.92	2	1	48.47	0	2.96
Molecule 8	408.92	2	1	48.47	0	2.96
Molecule 9	392.47	3	1	48.47	0	3.04
Molecule 10	392.47	3	1	48.47	0	3.06
Molecule 11	392.47	3	1	48.47	0	2.98
Molecule 12	388.51	2	1	48.47	0	3.08
Molecule 13	388.51	2	1	48.47	0	3.05
Molecule 14	388.51	2	1	48.47	0	3.02
Molecule 15	404.50	3	1	57.70	0	3.09
Molecule 16	404.50	3	1	57.70	0	3.10
Molecule 17	404.50	3	1	57.70	0	3.03
Molecule 18	399.49	3	1	72.26	0	3.08
Molecule 19	442.48	5	1	48.47	0	3.17
Molecule 20	410.46	4	1	48.47	0	3.11
Molecule 21	443.37	2	1	48.47	0	2.98
Molecule 22	410.46	4	1	48.47	0	3.08
Molecule 23	403.52	3	2	74.49	0	3.56
Molecule 24	417.55	3	2	74.49	0	3.65
Molecule 25	417.55	3	2	74.49	0	3.23
Molecule 26	445.60	3	2	74.49	0	3.42
Molecule 27	447.62	3	2	74.49	0	3.54
Molecule 28	473.65	3	2	74.49	0	4.16
Molecule 29	431.57	3	2	60.50	0	3.34
Molecule 30	445.60	3	1	51.71	0	3.46
Molecule 31	459.58	3	2	77.57	0	3.42
Molecule 32	418.53	3	2	68.70	0	3.19
Molecule 33	451.99	3	2	74.49	0	3.33
Molecule 34	451.99	3	2	74.49	0	3.31
Molecule 35	451.99	3	2	74.49	0	3.34

The blood-brain barrier (BBB) permeability is set at > 0.3 to −1, while the central nervous system (CNS) permeability is set at > −2 to −3. All of the top-hit compounds showed 100 % intestinal absorption in humans (HIA), suggesting that the human gut is capable of absorbing these anti-hepatitis C inhibitors. Additionally, the HIA values were intended to be higher than the minimum suggested rate of 30 % for the appraisal of this property. The hit compounds were found to have a BBB permeability of −1, suggesting that they might potentially penetrate or infiltrate the BBB. Since all substances have a CNS permeability value of −2, only extremely minute amounts of them can enter or permeate the central nervous system. The medications' metabolic characteristics are indicated by their proportionate action as CYP34A substrates and inhibitors. "Total clearance" describes the correlation between a drug's rate of excretion and its indicator concentration in the body.

Due to ADMET issues, many promising medications never make it to clinical trials. The number of compounds that failed clinical tests due to insufficient ADMET features has significantly decreased as a result of the importance of ADMET attributes, which are now being evaluated in the early phases of pharmacological studies (Daina et al., 2017). Human intestinal absorption (HIA), plasma glycoprotein (Pgp) substrate and inhibitor, blood-brain barrier (BBB) penetration, and human cytochromes (CYP) are among the key calculated ADMET parameters shown in Table 8. With expected values greater than −1, the lead compounds' BBB permeated output was categorized as BBB+. The predicted metabolic outputs showed that the likelihood of being either CYPs (1A2, 3A4, 2C9, 2C19, and 2D6) isoenzyme substrates or lead inhibitors varied from 0 to 1. Another important pharmacokinetic factor that describes how a drug is removed from the body is its clearance (CL). It is anticipated that the clearance penetration levels of the lead compounds will be low. Consequently, there were no significant respiratory or toxicity profile risks associated with any of the compounds.

**Table 8:** ADMET Properties of the best highest compounds.

S/N	GI Absorption	BBB permeant	Pgp substrate	Bioavailability score
Molecule 4	High	No	Yes	0.55
molecule 14	High	Yes	Yes	0.55
molecule 17	High	Yes	Yes	0.55
molecule 22	High	Yes	Yes	0.55
molecule 29	High	Yes	Yes	0.55

### 3.5 Structure‑based design of new Quinoline-2-yl (piperazin-1-yl) inhibitors

**Fig. 12:**
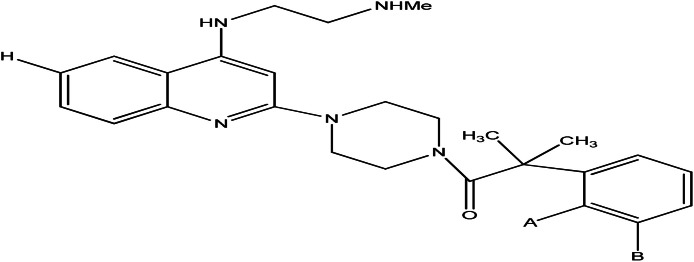
depicts the structural skeleton of compound 29 with A and B positions as points of connection.

**Fig. 13:**
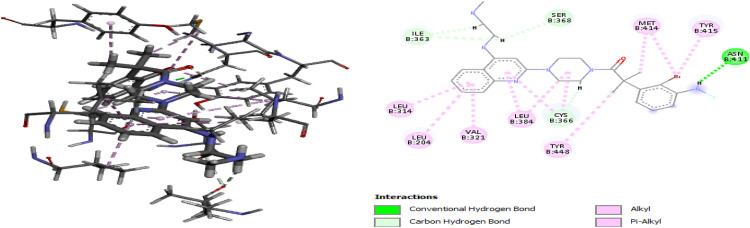
depicts the optimum SFBD3 design in 2D and 3D views, as well as hydrogen bond interactions, using the Discovery Studio virtualizer.

The A and B locations act as the connection points in Fig. 12, which shows how to create new molecules using compound 29 as a template. The newly designed receptor SFBD3′s docking compound in a binding pocket of NS5B polymerase exhibits exceptional docking scores (MolDock score: −152.299 kcal mol and Rerank score: −127.357 kcal mol), as shown in Table 9. Through ASN411 (2.19728 Å), CYS366 (2.73284 Å), ILE363 (3.05577 Å), SER368 (2.53969 Å), and ILE363 (2.19459 Å), the compound formed five conventional H-bonds and bond angles. The -NH group next to the pyridine ring formed a hydrophobic bond with CYS366 (4.41711 Å), LEU384 (5.0669 Å), and MET414 (3.42332 Å). MET414 (4.8011 Å), TYR415 (4.21448 Å), TYR448 (4.84263 Å), LEU204 (5.39891 Å), LEU314 (4.53829 Å), VAL321 (4.59218 Å), CYS366 (4.74862 Å), and LEU384 (5.08223 Å) all had -alkyl and Pi-alkyl interactions with their side chains. The study's findings indicate that the primary causes of inhibitor stability in the binding pocket are traditional H-bond interactions with crucial binding residues (Fig. 13, SCD3).

**Table 9:** shows the MolDockScore, Plants Score, and RerankScore for the newly designed compounds.

Name	MolDock Score kcal/mol.	Heavy Atoms	Plants Score	Rerank Score
SFBD 1	−95.5464	34	−68.2746	−76.8311
SFBD 2	−106.251	34	−74.1045	−88.3908
SFBD 3	−152.299	35	−82.7164	−127.357
SFBD 4	−147.363	35	−77.8605	−109.461
SFBD 5	−98.9696	35	−72.3714	−82.8459
SFBD 6	−138.63	34	−81.2727	−102.412

**Fig. 14:**
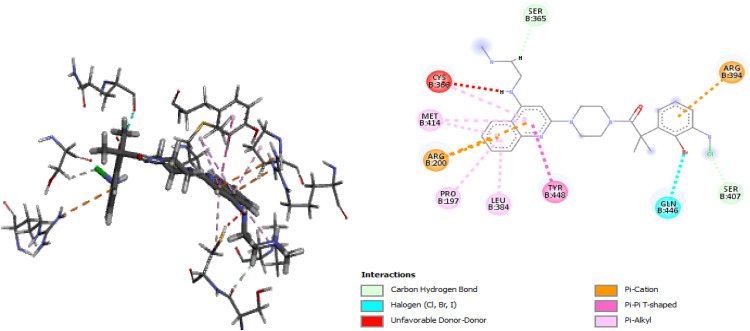
depicts the optimum SFBD4 design in 2D and 3D views, as well as hydrogen bond interactions, using the Discovery Studio virtualizer.

ARG200 (4.88678 Å) and ARG394 (4.57891 Å) form an electrostatic Pi-Cation with the benzene ring of the newly designed molecule SFBD4 (MolDock score −147.363 kcal mol and Rerank score −109.461 kcal mol), while TYR448 (4.79619 Å) forms a hydrophobic Pi-Pi T-shaped bond with the benzene ring. Additionally, the amine group combined with PRO197 (4.67823 Å), LEU384 (4.95232 Å), CYS366 (5.35062 Å), and MET414 (5.28511 Å) to create Hydrophobic Alkyl. The strong binding affinity may be explained by the hydrophobic and conventional H-bonding interactions between the NS5B polymerase and the ligand (Fig. 14, complex SFBD4).

The mole dock score of −152.299 and the Rerank score of −127.357 for all of the newly developed Quinoline-2-yl (piperazin-1-yl) Inhibitors are greater than those of the FDA-approved medication (Rib/Pig), which has a mole dock score of −111.095 and a Rerank score of −46.992.

### 3.6 Modeling and evaluating the ADME-toxic and drug-like properties of newly developed substances

Lipinski's rule of five was also used to model and assess the drug-likeness of recently produced compounds using the SwissADME web server. Originally, Lipinski's filters were used to analyze the drug-likeness of these novel compounds; both the modeled and assessed drug-likeness of the newly developed molecules showed no violations for any of the compounds. According to the drug-like screening criteria used in Table 10, the recently identified inhibitors have been identified as drug-like substances and are available orally. Furthermore, all of these can be easily created in the wet lab based on the synthetic accessibility characteristics. (Ibrahim et al., 2024).

**Table 10:** shows the MolDockScore, Plants Score, and RerankScore for the newly designed compounds.

Molecule	MW	H-bond acceptors	H-bond donors	TPSA	Lipinski violations	Synthetic Accessibility
SFBD 1	400.46	3	2	60.50	0	3.49
SFBD 2	444.91	3	2	60.50	0	3.53
SFBD 3	428.46	4	2	60.50	0	3.63
SFBD 4	443.47	4	3	72.53	0	3.78
SFBD 5	459.93	3	3	72.53	0	3.71
SFBD 6	443.47	4	3	72.53	0	3.78

According to Table 11, all of the produced compounds showed absorbance values higher than 30 %, showing outstanding absorbance in the typical human intestine (HI), demonstrating the newly synthesized ligands' considerable potential for barrier crossing. The newly created chemicals can pass through the blood-brain barrier, as evidenced by their penetration of < −1. Their penetration value into the central nervous system is much higher than −2. Proteins can therefore go through the central nervous system. The human body contains cytochrome (CYP450), a potent metabolic enzyme with five main isoforms: CYP A2, CYP2 C19, CYP2 C9, CYP2 D6, and CYP3 A4. The results displayed in Table 11 demonstrate that the proteins in question have acceptable pharmacokinetic interactions due to their potential inhibitory activity. The influence of a drug's dose and bioavailability in terms of achieving steady-state concentrations is measured by clearance. A drug ingredient can stay in the human body for extended periods of time if its clearance score is lower. The body reacted favorably to each of the recently created substances. To ascertain if a drug candidate is harmful or not, toxicity testing is performed. Table 11's results show that every newly created chemical was safe. Consequently, the selected compounds show the expected pharmacokinetic characteristics and may be used as prescription medications for hepatitis C. In summary, these recently created inhibitors' general ADME-Toxic properties showed good pharmacokinetic profiles and were shown to be better than the template.

**Table 11:** ADMET Properties of the newly designed compounds.

Molecule	GI absorption	BBB permeant	Pgp substrate	Bioavailability Score
SFBD 1	High	Yes	Yes	0.55
SFBD 2	High	Yes	Yes	0.55
SFBD 3	High	Yes	Yes	0.55
SFBD 4	High	Yes	Yes	0.55
SFBD 5	High	Yes	Yes	0.55
SFBD 6	High	Yes	Yes	0.55

## 4.0 Conclusion

In the pursuit of novel antiviral medications for the treatment of Hepatitis C virus (HCV) infections, the research presented in this study has successfully utilized advanced computational techniques to design and optimize Quinoline-2-yl (piperazin-1-yl) derivatives as potent inhibitors of the NS5B polymerase enzyme. The methodology employed a combination of 3D-QSAR modeling, DFT calculations, and molecular docking to predict the inhibitory effects of these compounds. The model developed through multilinear regression analysis and genetic function methods exhibited promising fitness values, indicating a strong predictive capacity for the inhibitory effects of the designed compounds against HCV. Validation techniques, including Applicability Domain analysis, VIF assessment, ME evaluation, and Y-scrambling test, confirmed the reliability and robustness of the model. Moreover, the pharmacokinetic properties and drug similarity of the Quinoline-2-yl (piperazin-1-yl) compounds were found to be favorable, suggesting their potential as effective anti-HCV agents. Molecular docking studies with the NS5B polymerase revealed that the compounds effectively interacted with the target protein, demonstrating their ability to occupy the binding sites and block the receptor. Furthermore, the creation of new analogues based on the highest docking score compound led to the identification of SFBD3 and SFBD4 as potent inhibitors of the NS5B polymerase, outperforming the FDA-approved medication Riv/peg. Molecular dynamics simulations evaluated the binding stability of compounds SFD B3 and 29 with HCV NS5B polymerase. Both compounds demonstrated stable active site associations, but SFD B3 exhibited superior conformational stability and stronger hydrogen bonding. These results highlight SFD B3 as a particularly promising candidate for novel anti-HCV agent development. These newly designed compounds showed enhanced docking scores, indicating improved interactions with the target protein, potentially enhancing their efficacy as antiviral agents.

## Permission

Permission has been given for use of copyrighted material from other sources (including the Internet).

## Funding

This research did not receive any specific grant from funding agencies in the public, commercial, or not-for-profit sectors.

## Compliance with ethics requirement

Not applicable.

## Research data

Data will be available on request

## CRediT authorship contribution statement

**Abubakar Sadiq Bello:** Writing – review & editing, Writing – original draft, Validation, Methodology, Conceptualization. **A. Uzairu:** Supervision, Conceptualization. **G.A Shallangwa:** Validation, Supervision, Conceptualization. **A. ibrahim:** Supervision, Conceptualization. **Mujeeb Khan:** Conceptualization, Methodology, Software. **M.T. Ibrahim:** Writing – review & editing, Supervision, Software, Methodology, Conceptualization.

## Declaration of competing interest

The authors declare that they have no known competing financial interests or personal relationships that could have appeared to influence the work reported in this paper.

## Data Availability

Data will be made available on request.
